# Post‐Marketing Surveillance of the Safety and Effectiveness of Cabozantinib in Japanese Patients With Advanced Renal Cell Carcinoma

**DOI:** 10.1111/iju.70549

**Published:** 2026-07-17

**Authors:** Hiro‐omi Kanayama, Shingo Kuroda, Tsuyoshi Osaka, Masatoshi Eto

**Affiliations:** ^1^ Department of Urology Kawashima Hospital Tokushima Japan; ^2^ Statistical & Quantitative Sciences, R&D Data & Quantitative Sciences Takeda Pharmaceutical Co. Ltd. Osaka Japan; ^3^ Japan Medical Affairs, Japan Oncology Business Unit Takeda Pharmaceutical Co. Ltd. Tokyo Japan; ^4^ Department of Urology, Graduate School of Medical Sciences Kyushu University Fukuoka Japan

**Keywords:** cabozantinib, effectiveness, nivolumab, post‐marketing surveillance, real‐world, renal cell carcinoma, safety

## Abstract

**Objectives:**

This post‐marketing surveillance study evaluated the real‐world safety and effectiveness of cabozantinib in Japanese patients with advanced renal cell carcinoma (aRCC).

**Methods:**

This prospective, observational study enrolled patients with histologically or cytologically confirmed aRCC across 102 sites in Japan. Patients received cabozantinib as monotherapy or in combination with nivolumab according to approved label regimens and were monitored for 26 weeks. The primary outcome was the incidence of adverse drug reactions (ADRs) related to hepatic failure/dysfunction and pancreatitis. Secondary outcomes included grade 3 or higher ADRs, treatment discontinuation due to ADRs, and investigator‐assessed objective response rate (ORR) using Response Evaluation Criteria in Solid Tumors (RECIST) version 1.1.

**Results:**

Of 388 enrolled patients, 385 were included in the analysis (median age, 71.0 years; 70.1% men). Patients receiving monotherapy (*n* = 322) and those receiving combination therapy (*n* = 63) reported ADRs related to hepatic failure/dysfunction (16.15% and 25.40%), pancreatitis‐related ADRs (2.80% and 4.76%), grade 3 or higher ADRs (24.84% and 38.10%), and ADRs leading to treatment discontinuation (13.98% and 30.16%), respectively. The most common grade 3 or higher ADRs were hypertension (3.73%), reduced appetite (3.11%), and hand‐foot syndrome (3.11%) in the monotherapy group, and diarrhea (4.76%) in the combination therapy group. The ORR was 32.9% for patients receiving monotherapy and 41.3% for those receiving combination therapy.

**Conclusions:**

Cabozantinib, as monotherapy or in combination with nivolumab, demonstrated safety and effectiveness in Japanese patients with aRCC in real‐world settings, consistent with previous literature. No new safety concerns were identified. These findings support the use of cabozantinib‐based regimens in Japanese clinical practice.

**Trail Registration:**

Japan Registry of Clinical Trials (JRCT), registration number: jRCT2031210003.

## 1 Introduction

Cabozantinib is an oral, small‐molecule tyrosine kinase inhibitor (TKI) that targets vascular endothelial growth factor receptor 2 (VEGFR2), MET, AXL, and other tyrosine kinases involved in tumor growth and metastasis [1]. Cabozantinib is approved as monotherapy for advanced renal cell carcinoma (aRCC), based on results from the global phase 3 METEOR trial and the Japanese phase 2 Cabozantinib‐2001 trial [2, 3, 4]. In the METEOR trial, cabozantinib demonstrated longer survival, delayed disease progression, and had a better tumor response compared with everolimus in previously treated patients with aRCC [2, 3]. The Cabozantinib‐2001 trial was designed to bridge the results from the METEOR trial to Japanese patients with aRCC who had been previously treated with a TKI [4]. In the CheckMate 9ER trial, the combination of cabozantinib with nivolumab, an immune checkpoint inhibitor, significantly improved survival outcomes compared with sunitinib in treatment‐naive patients with aRCC [5], leading to the approval of cabozantinib for combination therapy with nivolumab.

Although cabozantinib improves survival outcomes in patients with aRCC, it is associated with adverse drug reactions (ADRs), including diarrhea, fatigue, hypertension, decreased appetite, and hand‐foot syndrome [2, 3, 4, 5, 6]. Liver enzymes, such as alanine aminotransferase (ALT) or aspartate aminotransferase (AST), may also be elevated to more than three times the upper limit of normal with cabozantinib monotherapy and in combination with nivolumab [2, 3, 4, 5, 6]. Other abnormal safety signals may include increases in amylase and lipase, and pancreatitis or acute pancreatitis [4, 6].

While the safety profile of cabozantinib is well‐documented in the METEOR, Cabozantinib‐2001, and CheckMate 9ER studies, there are limited data on the safety and effectiveness of cabozantinib in Japanese patients with aRCC as well as on ADRs related to hepatic failure/dysfunction or pancreatitis. Therefore, this post‐marketing surveillance (PMS) study investigated the real‐world safety and effectiveness of cabozantinib in Japanese patients with aRCC, with a focus on the occurrence of hepatic failure or dysfunction, pancreatitis, and associated clinical laboratory values.

## 2 Methods

### 2.1 Study Design

This prospective, multicenter, observational PMS study evaluated the safety and effectiveness of cabozantinib in patients with aRCC (Japan Registry of Clinical Trials number: jRCT2031210003). The study enrolled patients from 102 centers in Japan through a central registration method using a web‐based electronic data capture system. The study was conducted in accordance with the Japanese Ministry Directive on Good Post‐Marketing Study Practice (GPSP), and the Ministry of Health, Labor, and Welfare of Japan approved the surveillance protocol. Informed consent was obtained. Ethics committee approvals were not required under the GPSP.

### 2.2 Patients and Treatment

Patients with histologically or cytologically confirmed aRCC were eligible for the study if they were about to start treatment with cabozantinib as monotherapy or in combination with nivolumab. Patients with a history of hypersensitivity to any component of cabozantinib were excluded. Patients received cabozantinib as monotherapy or in combination with nivolumab at doses determined by the physician's clinical assessment. Patients typically received cabozantinib 60 mg once daily (QD), 40 mg QD, 20 mg QD, or 20 mg every other day. Patients who commenced combination therapy received nivolumab 240 mg intravenously every 2 weeks in addition to their prescribed cabozantinib dose. Patient data were collected using case report forms. Patients were monitored from the first dose through 26 weeks or until treatment discontinuation. Patients were followed up until 30 days after discontinuation or until the initiation of subsequent systemic anti‐cancer therapy.

### 2.3 Outcomes

The primary outcome was the incidence of ADRs related to predefined safety specifications in the risk management plan for cabozantinib, namely hepatic failure, hepatic dysfunction, and pancreatitis. Hepatic failure/dysfunction was identified with the standardized MedDRA queries (SMQs) drug‐related hepatic dysfunction—severe events only (narrow), cholestasis and jaundice of hepatic origin (narrow), liver laboratory tests, signs and symptoms (narrow). Pancreatitis was identified with the SMQ acute pancreatitis (narrow) or the following preferred terms (PTs): amylase increased, amylase abnormal, lipase increased, lipase abnormal, pancreatic enzyme abnormality, pancreatic enzymes abnormal, pancreatic enzymes increased, hyperamylasemia, hyperlipasemia. After data collection, PTs were redistributed according to their respective primary system organ class (SOC).

Secondary outcomes included the incidence of grade 3 or higher ADRs (defined by the Common Terminology Criteria for Adverse Events version 5.0), ADRs leading to treatment discontinuation, and treatment effectiveness. Effectiveness was assessed by objective response rate (ORR; defined as the proportion of patients achieving a complete or partial response), as determined by investigators using the Response Evaluation Criteria in Solid Tumors (RECIST) version 1.1 criteria.

### 2.4 Sample Size and Statistical Analyses

Sample size calculations were informed by safety data from previous clinical trials. In the Cabozantinib‐2001 study, elevated ALT/AST levels occurred in approximately 20% of patients [4], supporting a target enrollment of 300 patients to yield at least 50 evaluable cases with hepatic dysfunction. The incidence of pancreatitis and acute pancreatitis ranged from 1.2% to 2.9% in earlier studies [3, 4], making this sample size sufficient to detect at least one case with 95% confidence. For combination therapy with nivolumab, elevated ALT/AST levels occurred in 31.8% of patients in the CheckMate 9ER study [5], justifying a target of 50 patients with at least 15 evaluable cases with hepatic dysfunction. The incidence of elevated pancreatic enzymes was also noted and supported this sample size. Given that liver‐related adverse events (AEs) typically occurred within 15 – 28 days, and pancreatitis‐related AEs around day 29 [3, 4], a 26‐week observation period was considered adequate to capture relevant ADRs.

Descriptive statistics were used to report patient baseline characteristics, ADRs related to hepatic failure/dysfunction and pancreatitis, grade 3 or higher ADRs, ADRs leading to treatment discontinuation, and tumor response. Subgroup analyses of ADRs related to hepatic failure/dysfunction and pancreatitis, and tumor response were stratified by patient baseline characteristics and initial treatment dose. No statistical comparisons were made. SAS version 9.4 was used for statistical analyses.

## 3 Results

### 3.1 Study Population

**FIGURE 1:**
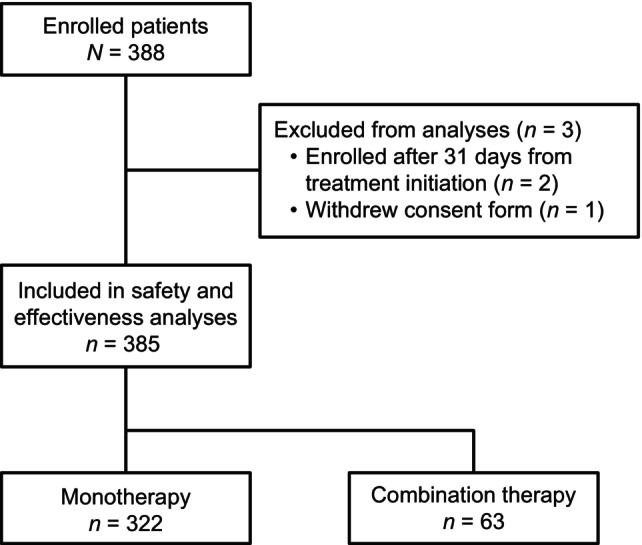
Patient disposition.

Case report forms were collected for 388 patients who were enrolled between March 2021 and August 2023 (Figure 1). Three patients were excluded from analyses owing to withdrawal of consent (*n* = 1) or enrollment after 31 days from treatment initiation (*n* = 2). In total, 385 patients were included in subsequent analyses.

Most patient demographics and baseline characteristics were similar between the treatment groups (Table 1 [7]). Overall, the median (range) age was 71.0 (20–90) years, 70.1% of patients were men, 81.8% of patients had a Karnofsky performance status of 80–100, and 85.5% had a total nephrectomy. More patients in the combination therapy group had non‐clear cell RCC (nccRCC) than in the monotherapy group (30.2% vs. 12.4%, respectively). Most patients had metastatic disease (97.7%); the most common metastasis site was lung (66.2%) for patients receiving monotherapy and bone (48.4%) for patients receiving combination therapy. Approximately half of the patients (50.6%) in the monotherapy group received cabozantinib as second‐line treatment.

**TABLE 1:** Patient demographics and baseline characteristics^a^.

	Monotherapy (*n* = 322)	Combination therapy (*n* = 63)	Overall (*n* = 385)
Male, *n* (%)	224 (69.6)	46 (73.0)	270 (70.1)
Age, years, median (range)	71.0 (20–90)	72.0 (35–87)	71.0 (20–90)
Karnofsky performance status, *n* (%)			
10–40	1 (0.3)	0 (0.0)	1 (0.3)
50–70	43 (13.4)	7 (11.1)	50 (13.0)
80–100	260 (80.7)	55 (87.3)	315 (81.8)
IMDC risk category, *n* (%)			
Favorable	68 (21.1)	18 (28.6)	86 (22.3)
Intermediate	175 (54.3)	35 (55.6)	210 (54.5)
Poor	64 (19.9)	10 (15.9)	74 (19.2)
Unknown	15 (4.7)	0 (0.0)	15 (3.9)
Histological type, *n* (%)			
ccRCC	264 (82.0)	42 (66.7)	306 (79.5)
nccRCC	40 (12.4)	19 (30.2)	59 (15.3)
Unknown	18 (5.6)	2 (3.2)	20 (5.2)
Had metastases, *n* (%)	314 (97.5)	62 (98.4)	376 (97.7)
Site of metastatic lesion, *n* (%)^b^			
Bone	121 (38.5)	30 (48.4)	151 (40.2)
Brain	23 (7.3)	6 (9.7)	29 (7.7)
Liver	49 (15.6)	11 (17.7)	60 (16.0)
Lung	208 (66.2)	27 (43.5)	235 (62.5)
Lymph node	100 (31.8)	13 (21.0)	113 (30.1)
Other	98 (31.2)	15 (24.2)	113 (30.1)
Previous treatment lines, *n* (%)			
0	46 (14.3)	—	—
1	163 (50.6)	—	—
2	57 (17.7)	—	—
3	36 (11.2)	—	—
≥ 4	20 (6.2)	—	—
Prior nephrectomy, *n* (%)			
Total nephrectomy	221 (86.3)	33 (80.5)	254 (85.5)
Partial nephrectomy	24 (9.4)	6 (14.6)	30 (10.1)
None	11 (4.3)	2 (4.9)	13 (4.4)
Medical history,^c^ *n* (%)	74 (23.0)	16 (25.4)	90 (23.4)
Had complications, *n* (%)	175 (54.3)	27 (42.9)	202 (52.5)
Had hepatic function disorder, *n* (%)	7 (2.2)	1 (1.6)	8 (2.1)
Had renal function disorder, *n* (%)	25 (7.8)	3 (4.8)	28 (7.3)

Abbreviations: ccRCC, clear cell renal cell carcinoma; IMDC, Internal Metastatic Renal Cell Carcinoma Database Consortium; nccRCC, non‐clear cell renal cell carcinoma.

^a^ Data are publicly available per local regulatory reporting requirements [7].

^b^ Only includes patients with metastases (monotherapy *n* = 314, combination therapy *n* = 62, overall *n* = 376). Patients may have more than one type of metastatic lesion.

^c^ Patients had a history of preoperative or postoperative adjuvant therapy prior to the first dose of cabozantinib.

### 3.2 Treatment Dosage, Modifications, and Discontinuation

In the monotherapy group, 100 patients (31.1%) started treatment on cabozantinib 60 mg QD, 145 patients (45.0%) on 40 mg QD, and 74 patients (23.0%) on 20 mg QD (Table 2 [7]). In the combination therapy group, two patients (3.2%) started treatment on cabozantinib 60 mg QD, 45 patients (71.4%) on 40 mg QD, and 15 patients (23.8%) on 20 mg QD. Dose modifications were required for patients in both treatment groups. For patients receiving monotherapy, 175 patients (54.3%) had a dose reduction, 135 patients (41.9%) had a dose interruption, and 120 patients (37.3%) discontinued treatment. For patients receiving combination therapy, 30 patients (47.6%) had a dose reduction, 22 patients (34.9%) had a dose interruption, and 33 patients (52.4%) discontinued treatment. The median (interquartile range) relative dose intensity of cabozantinib was 38.5% (33.3%–66.7%) for monotherapy and 61.6% (50.0%–100.0%) for combination therapy. The most common reason for treatment discontinuation in the monotherapy and combination therapy groups was AEs (*n* = 60 [50.0%] and *n* = 21 [63.6%], respectively), followed by disease progression (*n* = 42 [35.0%] and *n* = 7 [21.2%], respectively).

**TABLE 2:** Treatment dosage, modifications, and discontinuation^a^.

	Monotherapy (*n* = 322)	Combination therapy (*n* = 63)
Initial dose, *n* (%)		
60 mg QD	100 (31.1)	2 (3.2)
40 mg QD	145 (45.0)	45 (71.4)
20 mg QD	74 (23.0)	15 (23.8)
20 mg EOD	3 (0.9)	0 (0.0)
Other^b^	0 (0.0)	1 (1.6)
Dose, mg/day, median (range)	23.15 (3.9–60.0)	24.60 (3.3–40.7)
RDI, %, median (IQR)	38.5 (33.3–66.7)	61.6 (50.0–100.0)
Had dose reduction, *n* (%)	175 (54.3)	30 (47.6)
Had dose interruption, *n* (%)	135 (41.9)	22 (34.9)
Discontinued treatment, *n* (%)	120 (37.3)	33 (52.4)
Reason for treatment discontinuation, *n* (%)^c^		
Achieved treatment goal	2 (1.7)	3 (9.1)
AE	60 (50.0)	21 (63.6)
Lost to follow‐up	8 (6.7)	2 (6.1)
Pregnancy	0 (0.0)	0 (0.0)
Disease progression	42 (35.0)	7 (21.2)
Other	8 (6.7)	0 (0.0)

Abbreviations: AE, adverse event; EOD, every other day; IQR, interquartile range; QD, once daily; RDI, relative dose intensity.

^a^ Data are publicly available per local regulatory reporting requirements [7].

^b^ The other initial dose was cabozantinib 30 mg QD.

^c^ Only includes patients who discontinued treatment (monotherapy *n* = 120,; combination therapy *n* = 33).

### 3.3 Hepatic Failure/Dysfunction and Pancreatitis

In total, 52 patients (16.15%) receiving monotherapy reported ADRs relating to hepatic failure/dysfunction, with two patients (0.62%) experiencing serious ADRs: liver function abnormality (*n* = 1) and abnormal liver function test (*n* = 1) (Table 3). Of the 52 patients, 28 (53.85%) had recovered and 24 (46.15%) had improved by week 26. In total, 16 patients (25.40%) receiving combination therapy reported ADRs relating to hepatic failure/dysfunction, with four patients (6.35%) experiencing serious ADRs: liver function abnormality, hepatic disorder, immune‐mediated hepatic disorder, increased ALT, and increased AST (*n* = 1 each). Of the 16 patients, nine (56.25%) had recovered, six (37.50%) had improved, and one (6.25%) had not recovered by week 26.

**TABLE 3:** Incidence of predefined safety specifications related to hepatic failure/dysfunction and pancreatitis.

Predefined safety specifications, *n* (%)	Monotherapy (*n* = 322)		Combination therapy (*n* = 63)	
	Total ADRs	Serious ADRs^a^	Total ADRs	Serious ADRs^a^
Hepatic failure/dysfunction	52 (16.15)	2 (0.62)	16 (25.40)	4 (6.35)
Hepatobiliary disorders	33 (10.25)	1 (0.31)	12 (19.05)	3 (4.76)
Liver function abnormality	28 (8.70)	1 (0.31)	7 (11.11)	1 (1.59)
Liver disorders	5 (1.55)	0 (0.00)	4 (6.35)	1 (1.59)
Immune‐mediated hepatic disorder	0 (0.00)	0 (0.00)	1 (1.59)	1 (1.59)
Investigations	19 (5.90)	1 (0.31)	4 (6.35)	1 (1.59)
Increased ALT	10 (3.11)	0 (0.00)	3 (4.76)	1 (1.59)
Increased AST	7 (2.17)	0 (0.00)	3 (4.76)	1 (1.59)
Increased GGT	1 (0.31)	0 (0.00)	0 (0.00)	0 (0.00)
Abnormal liver function test	3 (0.93)	1 (0.31)	1 (1.59)	0 (0.00)
Elevated liver enzymes	2 (0.62)	0 (0.00)	0 (0.00)	0 (0.00)
Increased liver function test values	2 (0.62)	0 (0.00)	0 (0.00)	0 (0.00)
Pancreatitis	9 (2.80)	4 (1.24)	3 (4.76)	1 (1.59)
Gastrointestinal disorders	3 (0.93)	3 (0.93)	1 (1.59)	1 (1.59)
Pancreatitis	2 (0.62)	2 (0.62)	0 (0.00)	0 (0.00)
Acute pancreatitis	1 (0.31)	1 (0.31)	1 (1.59)	1 (1.59)
Investigations	6 (1.86)	1 (0.31)	2 (3.17)	0 (0.00)
Increased amylase	5 (1.55)	1 (0.31)	1 (1.59)	0 (0.00)
Increased lipase	2 (0.62)	1 (0.31)	2 (3.17)	0 (0.00)

*Note:* Predefined safety specifications are listed in hierarchical order of standardized MedDRA query, system organ class, and preferred term.

Abbreviations: ADR, adverse drug reaction; ALT, alanine aminotransferase; AST, aspartate aminotransferase; GGT, gamma‐glutamyl transferase.

^a^ An ADR was considered serious if it was life‐threatening, caused significant disability/incapacity, led to hospitalization, or led to death.

Nine patients (2.80%) who received monotherapy experienced ADRs related to pancreatitis, with four patients (1.24%) experiencing serious ADRs: pancreatitis (*n* = 2), acute pancreatitis (*n* = 1), increased amylase (*n* = 1), and increased lipase (*n* = 1). Of the nine patients, five (55.56%) had recovered, three (33.33%) had improved, and one (1.11%) had an unknown outcome by week 26. Three patients (4.76%) who received combination therapy experienced ADRs related to pancreatitis, with one patient (1.59%) experiencing a serious ADR of acute pancreatitis. Of the three patients, one (33.33%) had recovered and two (66.67%) had improved by week 26.

Similar incidences of abnormal hepatic laboratory tests were observed in the monotherapy (5.90%) and combination therapy (6.35%) groups. In the monotherapy group, increased ALT and AST were observed in 10 patients (3.11%) and seven patients (2.17%), respectively. In the combination therapy group, increased ALT and AST were each observed in three patients (4.76%). Six patients (1.86%) receiving monotherapy and two patients (3.17%) receiving combination therapy had elevated pancreatic enzymes.

The median time to first onset of hepatic failure/dysfunction ADRs was 15.0 and 29.5 days in patients receiving monotherapy and combination therapy, respectively. The median time to first onset of pancreatitis in the monotherapy and combination therapy groups was 16.0 and 63.0 days, respectively.

### 3.4 Other Safety Outcomes

Overall, 80 patients (24.84%) receiving monotherapy and 24 patients (38.10%) receiving combination therapy reported an ADR of grade 3 or higher (Table 4). Of these, the most common were hypertension (*n* = 12 [3.73%]), reduced appetite, and hand‐foot syndrome (*n* = 10 [3.11%] each) in the monotherapy group and diarrhea (*n* = 3 [4.76%]) in the combination therapy group.

**TABLE 4:** Incidence of grade 3 or higher ADRs.

	Monotherapy (*n* = 322)	Combination therapy (*n* = 63)
Any ADR, *n* (%)	80 (24.84)	24 (38.10)
ADRs reported in ≥ 2 patients, *n* (%)		
Hypertension	12 (3.73)	1 (1.59)
Reduced appetite	10 (3.11)	1 (1.59)
Hand‐foot syndrome	10 (3.11)	2 (3.17)
Proteinuria	8 (2.48)	0 (0.00)
Diarrhea	6 (1.86)	3 (4.76)
Malaise	5 (1.55)	0 (0.00)
Anemia	4 (1.24)	0 (0.00)
Liver function abnormality	3 (0.93)	2 (3.17)
Hypothyroidism	3 (0.93)	0 (0.00)
Stomatitis	3 (0.93)	0 (0.00)
Renal impairment	3 (0.93)	0 (0.00)
Chronic kidney disease	3 (0.93)	0 (0.00)
Hyponatremia	2 (0.62)	0 (0.00)
Interstitial lung disease	2 (0.62)	2 (3.17)
Increased ALT	2 (0.62)	2 (3.17)
Decreased neutrophil count	1 (0.31)	2 (3.17)
Liver disorder	1 (0.31)	2 (3.17)
Increased AST	0 (0.00)	2 (3.17)
Increased lipase	1 (0.31)	2 (3.17)

Abbreviations: ADR, adverse drug reaction; ALT, alanine aminotransferase; AST, aspartate aminotransferase.

In the monotherapy group, ADRs led to dose reductions in 112 patients (34.78%), dose interruptions in 91 patients (28.26%), and treatment discontinuations in 45 patients (13.98%) (Table 5). In the combination therapy group, ADRs led to dose reductions in 18 patients (28.57%), dose interruptions in 14 patients (22.22%), and treatment discontinuations in 19 patients (30.16%). The most common ADRs that led to dose reduction and dose interruption in the monotherapy group were hand‐foot syndrome (7.45% and 5.28%, respectively) and liver function abnormality (5.90% and 4.66%, respectively), whereas reduced appetite was the most common ADR leading to treatment discontinuation (3.42%). In the combination therapy group, hand‐foot syndrome and liver function abnormality were also the most common ADRs leading to dose reduction (6.35% and 7.94%, respectively), and hand‐foot syndrome was the most common ADR for dose interruption (4.76%). Reduced appetite, interstitial lung disease, diarrhea, and rash equally led to treatment discontinuation in the combination therapy group (3.17% each).

**TABLE 5:** Proportion of patients with ADRs that led to dose modification.

ADRs	Monotherapy (*n* = 322)	Combination therapy (*n* = 63)
ADRs that led to dose reduction,^a^ *n* (%)	112 (34.78)	18 (28.57)
Hand‐foot syndrome	24 (7.45)	4 (6.35)
Liver function abnormality	19 (5.90)	5 (7.94)
Diarrhea	13 (4.04)	2 (3.17)
Hypertension	12 (3.73)	1 (1.59)
Reduced appetite	9 (2.80)	0 (0.00)
Malaise	8 (2.48)	0 (0.00)
Decreased neutrophil count	1 (0.31)	2 (3.17)
ADRs that led to dose interruption,^a^ *n* (%)	91 (28.26)	14 (22.22)
Hand‐foot syndrome	17 (5.28)	3 (4.76)
Liver function abnormality	15 (4.66)	2 (3.17)
Diarrhea	14 (4.35)	0 (0.00)
Proteinuria	10 (3.11)	0 (0.00)
Drug eruption	1 (0.31)	2 (3.17)
Increased lipase	1 (0.31)	2 (3.17)
Increased AST	5 (1.55)	2 (3.17)
ADRs that led to drug discontinuation,^b^ * n* (%)	45 (13.98)	19 (30.16)
Reduced appetite	11 (3.42)	2 (3.17)
Malaise	6 (1.86)	0 (0.00)
Interstitial lung disease	3 (0.93)	2 (3.17)
Stomatitis	3 (0.93)	0 (0.00)
Diarrhea	2 (0.62)	2 (3.17)
Rash	1 (0.31)	2 (3.17)

Abbreviations: ADR, adverse drug reaction; AST, aspartate aminotransferase.

^a^ Individual ADRs reported in at least 2% of patients for monotherapy and at least two patients for combination therapy.

^b^ Individual ADRs reported in at least three patients for monotherapy and at least two patients for combination therapy.

The incidence of ADRs was relatively consistent across patient demographics and baseline characteristics (Table S1). A dose‐proportional trend was observed, with higher initial dosages associated with more ADRs than lower or less frequent dosages.

### 3.5 Tumor Response

Investigator‐assessed ORR was 32.9% (95% confidence interval [CI] 27.81–38.35) for patients receiving monotherapy and 41.3% (95% CI 29.01–54.38) for patients receiving combination therapy. Tumor response stratified by patient characteristics and treatment dose is shown in Table S2. For both treatment groups, the ORR was greatest for patients with clear cell RCC, favorable Internal Metastatic Renal Cell Carcinoma Database Consortium risk category, lung metastasis, and an initial cabozantinib dose of 60 mg QD. For patients with nccRCC, the ORR was 15.0% (95% CI 5.71–29.84) for patients receiving monotherapy versus 36.8% (95% CI 16.29–61.64) for patients receiving combination therapy.

## 4 Discussion

This prospective, observational study demonstrated the safety and effectiveness of cabozantinib monotherapy or combination therapy with nivolumab in patients with aRCC under routine clinical practice. The safety and tolerability of cabozantinib in this PMS study were consistent with previously reported cabozantinib data and the safety profile of other TKIs in Japanese patients [3, 4, 5, 8, 9, 10]. While hypertension, reduced appetite, and hand‐foot syndrome were the most common grade 3 or higher ADRs in the monotherapy group, diarrhea was the most common grade 3 or higher ADR in the combination therapy group, which is consistent with the safety profile of nivolumab [11].

It is well‐known that cabozantinib can cause elevations in liver enzymes, and as an immune checkpoint inhibitor that enhances the immune response, nivolumab may contribute to these elevations and also to hepatotoxicity [11, 12, 13]. Consistent with this, hepatic failure/dysfunction occurred in 16.15% of patients receiving cabozantinib monotherapy and 25.40% of patients receiving combination therapy with nivolumab. The incidence of elevated ALT and AST related to hepatic failure/dysfunction was 3.11% and 2.17% in the monotherapy group, respectively, and 4.76% for each enzyme in the combination therapy group, of which one case was deemed serious for each enzyme. The overall incidence of grade 3 or higher ADRs of ALT or AST elevations with cabozantinib monotherapy was lower in our study (0.62% and 0.00%, respectively) compared with the METEOR trial (1.8% and 0.9%, respectively) [14].

In our study, the incidence of pancreatitis‐related ADRs for patients receiving cabozantinib monotherapy (2.80%; pancreatitis: 0.62%, acute pancreatitis: 0.31%, increased amylase: 1.55%, increased lipase: 0.62%) was comparable to or lower than the incidence of pancreatitis‐related ADRs from the METEOR trial (pancreatitis: 0.6%, acute pancreatitis: 0.3%, increased amylase: 3.3%, increased lipase: 2.7%) and Cabozantinib‐2001 trial (pancreatitis: 0%, acute pancreatitis: 0%, increased amylase: 8.6%, increased lipase: 5.7%, pancreatic enzymes increased 2.9%) [14]; however, the rate was slightly higher for patients receiving combination therapy (4.76%), possibly owing to nivolumab's immune‐mediated effects [11].

In clinical trials, most patients typically commenced treatment with cabozantinib 60 mg QD as monotherapy or cabozantinib 40 mg QD as combination therapy with nivolumab, which are the approved cabozantinib starting doses in Japan [2, 3, 4, 5, 10, 15]. In our study, almost 70% of patients started cabozantinib monotherapy at a dose lower than 60 mg QD, and more patients in both treatment groups initiated cabozantinib 40 mg QD compared with other dosages. This difference from clinical trials may reflect real‐world treatment decisions tailored to patient‐specific factors, such as disease histology, treatment history, and age. Compared with clinical trials, real‐world populations often include older patients with poorer prognostic profiles, lower Karnofsky performance scores, and a greater burden of comorbidities that can affect treatment selection and outcomes. Notably, 15.3% of patients in our study had nccRCC, and most patients (85.7%) in the monotherapy group had received previous lines of treatment. Additionally, patients were 20 to 90 years old with a median age of 71 years, which was higher than the median age reported in the METEOR, Cabozantinib‐2001, CABOSUN, and CheckMate 9ER trials (62–63 years) [2, 3, 4, 5, 10]. Furthermore, a greater proportion of patients in the METEOR trial had a favorable prognostic profile compared with patients in our study (45% vs. 21%), although a different prognostic risk profile was used versus our study [2, 3]. Our findings reflect real‐world prescribing practice and should not be interpreted as supporting deviation from the labeled initiation dose. Cabozantinib should be initiated at the recommended labeled dose of 60 mg QD, with dose reductions applied as clinically appropriate [15].

A higher proportion of patients in our study discontinued treatment owing to ADRs compared with previous reports [3, 4, 5]. While ADRs led to treatment discontinuation in 13.98% of patients receiving cabozantinib monotherapy in our study, the METEOR and Cabozantinib‐2001 trials reported discontinuation rates of 12.08% and 5.71%, respectively, owing to AEs [3, 4]. For combination therapy, 30.16% of patients in our study discontinued treatment owing to ADRs, whereas in the CheckMate 9ER trial, ADRs leading to discontinuation of at least one study drug were observed in 15.3% of patients [16]. These differences may also reflect inherent distinctions between clinical trials and real‐world studies such as motivation, treatment adherence, management of AEs, disease progression, disease severity, and comorbidities.

The results of our study support previously published data on the efficacy of cabozantinib in treating RCC [4, 5, 17]. In our study, the ORR for patients receiving monotherapy (32.9%) was higher than that reported in the METEOR and Cabozantinib‐2001 trials (17% and 20%, respectively) and similar to the CABOSUN trial (33%) [3, 4, 10]. However, the ORR was lower for patients receiving combination therapy compared with the CheckMate 9ER trial (41.3% vs. 55.7%) [5]. The lower ORR in our study may reflect the inclusion of more patients with poor prognosis, such as those with nccRCC or bone metastases. On average, 25% of RCC cases are nccRCC [18]; however, almost one‐third of patients who received combination therapy in our study had nccRCC. Although nccRCC comprises diverse tumor types that can be difficult to treat [18], cabozantinib plus nivolumab has shown promising efficacy in treating papillary, unclassified, and translocation‐associated nccRCC with an ORR of 47.5% [19]. This is supported by our subgroup analysis in which patients with nccRCC who received cabozantinib with nivolumab had an ORR of 36.8%. Nevertheless, comparisons of outcomes across studies should be approached with caution owing to variations in study design, patient populations, protocols, and other influencing factors.

Our study has some limitations that may contribute to certain differences between our findings and those of previous trials, such as including patients without targeted lesions as per RECIST, lower starting doses of cabozantinib, and reliance on investigator assessments without independent central review. Investigator assessments without independent central review may have introduced inter‐observer variability and differences in the timing of tumor assessments across institutions. This may have influenced response classification and confirmation, potentially resulting in over‐ or underestimation of ORR compared with centrally reviewed clinical trials. Another limitation of our study was that time to improvement was not assessed for ADRs related to hepatic and/or pancreatic dysfunction, which may be clinically relevant and warrants evaluation in future studies. Additionally, information on the reasons for cabozantinib dose reductions was not available; therefore, limiting insight into the clinical decision‐making underlying dose modifications.

Overall, this real‐world PMS study supports the use of cabozantinib, as monotherapy or in combination with nivolumab, for treating Japanese patients with aRCC in clinical practice. The incidence of ADRs related to hepatic failure/dysfunction was higher than pancreatitis‐related ADRs. No new safety concerns were identified in either treatment group over the 6‐month observation period. Nonetheless, clinicians should consider the risks and benefits of modifying cabozantinib doses for patients with aRCC.

## Author Contributions

**Hiro‐omi Kanayama:** conceptualization, data curation, investigation, methodology, writing – review and editing. **Shingo Kuroda:** conceptualization, formal analysis, investigation, methodology, writing – review and editing. **Tsuyoshi Osaka:** conceptualization, formal analysis, investigation, methodology, project administration, resources, writing – original draft, writing – review and editing. **Masatoshi Eto:** conceptualization, data curation, investigation, methodology, writing – review and editing.

## Funding

This study was funded by Takeda Pharmaceutical Company and Limited.

## Ethics Statement

The study was conducted in accordance with the Japanese Ministry Directive on Good Post‐Marketing Study Practice (GPSP), and the Ministry of Health, Labor, and Welfare of Japan approved the surveillance protocol. Ethics committee approvals were not required under the GPSP.

## Consent

Informed consent was obtained.

## Conflicts of Interest

Hiro‐omi Kanayama has received honoraria for expert testimony and meeting/travel fees from Takeda Pharmaceutical Co. Ltd. Shingo Kuroda and Tsuyoshi Osaka are employees of and have stock ownership with Takeda Pharmaceutical Co. Ltd. Masatoshi Eto has received research funding from MSD and speaker fees from Astellas Pharma, AstraZeneca, Bayer Yakuhin, Bristol Myers Squibb, Eisai, Janssen Pharmaceutical, Merck Biopharma, MSD, Novartis, Ono Pharmaceutical, Pfizer, and Takeda Pharmaceutical Co. Ltd.

## Supporting information


**Table S1:** Incidence of ADRs by patient baseline characteristics and initial treatment dose.
**Table S2:** Investigator‐assessed tumor responses by patient baseline disease characteristics and initial treatment dose (using RECIST v1.1).

## Data Availability

The datasets, including the redacted study protocol, redacted statistical analysis plan, and individual participants data supporting the results reported in this article, will be made available within three months from initial request, to researchers who provide a methodologically sound proposal. The data will be provided after its de‐identification, in compliance with applicable privacy laws, data protection and requirements for consent and anonymization.
