# Supplementary material for: Post‐Marketing Surveillance of the Safety and Effectiveness of Cabozantinib in Japanese Patients With Advanced Renal Cell Carcinoma
Source: Int J Urol. 2026 Jul 17;33(7):e70549. doi: 10.1111/iju.70549 (PMC13376992; doi:10.1111/iju.70549)
Supplement: Supplementary file 1 — Table S1: Incidence of ADRs by patient baseline characteristics and initial treatment dose. Table S2: Investigator‐assessed tumor responses by patient baseline disease characteristics and initial treatment dose (using RECIST v1.1). [file IJU-33-0-s001.docx]

# **Post-Marketing Surveillance of the Safety And Effectiveness of Cabozantinib In Japanese Patients With Advanced Renal Cell Carcinoma**

Hiro-omi Kanayama,^1^ Shingo Kuroda,^2^ Tsuyoshi Osaka,^3^ Masatoshi Eto^4^

**Affiliations**

^1^Department of Urology, Kawashima Hospital, Tokushima, Japan

^2^Statistical & Quantitative Sciences, R&D Data & Quantitative Sciences, Takeda Pharmaceutical Co. Ltd., Osaka, Japan

^3^Japan Medical Affairs, Japan Oncology Business Unit, Takeda Pharmaceutical Co. Ltd., Tokyo, Japan

^4^Department of Urology, Graduate School of Medical Sciences, Kyushu University, Fukuoka, Japan

**Corresponding author**

Hiro-omi Kanayama

Department of Urology, Kawashima Hospital, 6-1 Kitasakoichiban-cho, Tokushima, 770-8548, Japan

**E-mail:** kanayama.hiroomi@arrow.ocn.ne.jp

**Phone:** +81-88-631-0110

**Fax:** +81-88-631-5500

**Supporting Information**

**TABLE S1.** Incidence of ADRs by patient baseline characteristics and initial treatment dose.

| **Characteristic** | ***n*** | **Total ADRs,**  ***n* (%)** | **Hepatic failure, hepatic function disorder, *n* (%)** | **Pancreatitis, *n* (%)** |
| --- | --- | --- | --- | --- |
| **Sex** | | | | |
| Male | 270 | 160 (59.26) | 45 (16.67) | 9 (3.33) |
| Female | 115 | 74 (64.35) | 23 (20.00) | 3 (2.61) |
| **Age, years** | | | | |
| 18–64 | 112 | 65 (58.04) | 22 (19.64) | 1 (0.89) |
| 65–74 | 140 | 89 (63.57) | 25 (17.86) | 3 (2.14) |
| 75–84 | 112 | 69 (61.61) | 19 (16.96) | 8 (7.14) |
| ≥85 | 21 | 11 (52.38) | 2 (9.52) | 0 (0.00) |
| **Karnofsky performance status** | | | | |
| 10–40 | 1 | 0 (0.00) | 0 (0.00) | 0 (0.00) |
| 50–70 | 50 | 29 (58.00) | 8 (16.00) | 2 (4.00) |
| 80–100 | 315 | 192 (60.95) | 56 (17.78) | 9 (2.86) |
| **IMDC risk category** | | | | |
| Favorable | 86 | 56 (65.12) | 21 (24.42) | 2 (2.33) |
| Intermediate | 210 | 126 (60.00) | 32 (15.24) | 8 (3.81) |
| Poor | 74 | 39 (52.70) | 10 (13.51) | 2 (2.70) |
| Unknown | 15 | 13 (86.67) | 5 (33.33) | 0 (0.00) |
| **Histological type** | | | | |
| Clear cell | 306 | 189 (61.76) | 55 (17.97) | 10 (3.27) |
| Non-clear cell | 59 | 34 (57.63) | 9 (15.25) | 2 (3.39) |
| Unknown | 20 | 11 (55.00) | 4 (20.00) | 0 (0.00) |
| **Medical history^a^** | | | | |
| Yes | 90 | 60 (66.67) | 13 (14.44) | 6 (6.67) |
| No | 295 | 174 (58.98) | 55 (18.64) | 6 (2.03) |
| **Complications** | | | | |
| Yes | 202 | 128 (63.37) | 44 (21.78) | 7 (3.47) |
| No | 183 | 106 (57.92) | 24 (13.11) | 5 (2.73) |

| **Hepatic function disorder** | | | | |
| --- | --- | --- | --- | --- |
| Yes | 8 | 6 (75.00) | 2 (25.00) | 0 (0.00) |
| No | 377 | 228 (60.48) | 66 (17.51) | 12 (3.18) |
| **Renal function disorder** | | | | |
| Yes | 28 | 13 (46.43) | 3 (10.71) | 1 (3.57) |
| No | 357 | 221 (61.90) | 65 (18.21) | 11 (3.08) |
| **Initial cabozantinib dose** | | | | |
| 60 mg QD | 102 | 70 (68.63) | 24 (23.53) | 3 (2.94) |
| 40 mg QD | 190 | 124 (65.26) | 37 (19.47) | 7 (3.68) |
| 20 mg QD | 89 | 40 (44.94) | 7 (7.87) | 2 (2.25) |
| 20 mg EOD | 3 | 0 (0.00) | 0 (0.00) | 0 (0.00) |
| Other^a^ | 1 | 0 (0.00) | 0 (0.00) | 0 (0.00) |

^a^Patients had a history of preoperative or postoperative adjuvant therapy prior to the first dose of cabozantinib.

^b^The other initial dose was cabozantinib 30 mg QD.

ADR, adverse drug reaction; EOD, every other day; IMDC, Internal Metastatic Renal Cell Carcinoma Database Consortium; QD, once daily.

**TABLE S2.** Investigator-assessed tumor responses by patient baseline disease characteristics and initial treatment dose (using RECIST version 1.1).

|  | | | ***n* (%; 95% CI)** |  | ***n* (%)** | | | | | |
| --- | --- | --- | --- | --- | --- | --- | --- | --- | --- | --- |
|  | | ***n*** | **ORR** | **CR** | **PR** | **SD** | **PD** | **NE** | **Non-CR/**  **non-PD** | **Missing** |
| **Monotherapy** | | | | | | | | | | |
| Overall | | 322 | 106 (32.9; 27.81–38.35) | 4 (1.2) | 102 (31.7) | 132 (41.0) | 46 (14.3) | 12 (3.7) | 9 (2.8) | 17 (5.3) |
| Histological type | Clear cell | 264 | 96 (36.4; 30.55–42.48) | 3 (1.1) | 93 (35.2) | 103 (39.0) | 31 (11.7) | 10 (3.8) | 9 (3.4) | 15 (5.7) |
|  | Non-clear cell | 40 | 6 (15.0; 5.71–29.84) | 0 (0.0) | 6 (15.0) | 20 (50.0) | 11 (27.5) | 1 (2.5) | 0 (0.0) | 2 (5.0) |
| IMDC risk category | Favorable | 68 | 30 (44.1; 32.08–56.68) | 2 (2.9) | 28 (41.2) | 28 (41.2) | 5 (7.4) | 2 (2.9) | 0 (0.0) | 3 (4.4) |
|  | Intermediate | 175 | 64 (36.6; 29.43–44.17) | 2 (1.1) | 62 (35.4) | 67 (38.3) | 23 (13.1) | 8 (4.6) | 5 (2.9) | 8 (4.6) |
|  | Poor | 64 | 12 (18.8; 10.08–30.46) | 0 (0.0) | 12 (18.8) | 23 (35.9) | 18 (28.1) | 1 (1.6) | 4 (6.3) | 6 (9.4) |
| Site of metastatic lesion | Brain | 23 | 5 (21.7; 7.46–43.70) | 0 (0.0) | 5 (21.7) | 6 (26.1) | 4 (17.4) | 2 (8.7) | 2 (8.7) | 4 (17.4) |
|  | Lung | 208 | 77 (37.0; 30.44–43.97) | 2 (1.0) | 75 (36.1) | 77 (37.0) | 28 (13.5) | 8 (3.8) | 6 (2.9) | 12 (5.8) |
|  | Liver | 49 | 13 (26.5; 14.95–41.08) | 0 (0.0) | 13 (26.5) | 18 (36.7) | 12 (24.5) | 3 (6.1) | 1 (2.0) | 2 (4.1) |
|  | Bone | 121 | 28 (23.1; 15.96–31.68) | 1 (0.8) | 27 (22.3) | 54 (44.6) | 22 (18.2) | 4 (3.3) | 6 (5.0) | 7 (5.8) |
|  | Lymph node | 100 | 29 (29.0; 20.36–38.93) | 0 (0.0) | 29 (29.0) | 46 (46.0) | 15 (15.0) | 2 (2.0) | 3 (3.0) | 5 (5.0) |
| Initial cabozantinib dose | 60 mg QD | 100 | 43 (43.0; 33.14–53.29) | 2 (2.0) | 41 (41.0) | 40 (40.0) | 9 (9.0) | 3 (3.0) | 2 (2.0) | 3 (3.0) |
|  | 40 mg QD | 145 | 42 (29.0; 21.74–37.07) | 2 (1.4) | 40 (27.6) | 62 (42.8) | 23 (15.9) | 5 (3.4) | 5 (3.4) | 8 (5.5) |
|  | 20 mg QD | 74 | 20 (27.0; 17.35–38.61) | 0 (0.0) | 20 (27.0) | 29 (39.2) | 14 (18.9) | 4 (5.4) | 1 (1.4) | 6 (8.1) |
|  | 20 mg EOD | 3 | 1 (33.3; 0.84–90.57) | 0 (0.0) | 1 (33.3) | 1 (33.3) | 0 (0.0) | 0 (0.0) | 1 (33.3) | 0 (0.0) |

| **Combination therapy** | | | | | | | | | | |
| --- | --- | --- | --- | --- | --- | --- | --- | --- | --- | --- |
| Overall | | 63 | 26 (41.3; 29.01–54.38) | 2 (3.2) | 24 (38.1) | 28 (44.4) | 7 (11.1) | 1 (1.6) | 0 (0.0) | 1 (1.6) |
| Histological type | Clear cell | 42 | 19 (45.2; 29.85–61.33) | 0 (0.0) | 19 (45.2) | 18 (42.9) | 4 (9.5) | 0 (0.0) | 0 (0.0) | 1 (2.4) |
|  | Non-clear cell | 19 | 7 (36.8; 16.29–61.64) | 2 (10.5) | 5 (26.3) | 8 (42.1) | 3 (15.8) | 1 (5.3) | 0 (0.0) | 0 (0.0) |
| IMDC risk category | Favorable | 18 | 11 (61.1; 35.75–82.70) | 0 (0.0) | 11 (61.1) | 7 (38.9) | 0 (0.0) | 0 (0.0) | 0 (0.0) | 0 (0.0) |
|  | Intermediate | 35 | 15 (42.9; 26.32–60.65) | 2 (5.7) | 13 (37.1) | 15 (42.9) | 4 (11.4) | 0 (0.0) | 0 (0.0) | 1 (2.9) |
|  | Poor | 10 | 0 (0.0; 0.00–30.85) | 0 (0.0) | 0 (0.0) | 6 (60.0) | 3 (30.0) | 1 (10.0) | 0 (0.0) | 0 (0.0) |
| Site of metastatic lesion | Brain | 6 | 1 (16.7; 0.42–64.12) | 0 (0.0) | 1 (16.7) | 3 (50.0) | 1 (16.7) | 0 (0.0) | 0 (0.0) | 1 (16.7) |
|  | Lung | 27 | 12 (44.4; 25.48–64.67) | 0 (0.0) | 12 (44.4) | 12 (44.4) | 2 (7.4) | 0 (0.0) | 0 (0.0) | 1 (3.7) |
|  | Liver | 11 | 2 (18.2; 2.28–51.78) | 0 (0.0) | 2 (18.2) | 7 (63.6) | 2 (18.2) | 0 (0.0) | 0 (0.0) | 0 (0.0) |
|  | Bone | 30 | 8 (26.7; 12.28–45.89) | 1 (3.3) | 7 (23.3) | 17 (56.7) | 3 (10.0) | 1 (3.3) | 0 (0.0) | 1 (3.3) |
|  | Lymph node | 13 | 3 (23.1; 5.04–53.81) | 0 (0.0) | 3 (23.1) | 5 (38.5) | 3 (23.1) | 1 (7.7) | 0 (0.0) | 1 (7.7) |
| Initial cabozantinib dose | 60 mg QD | 2 | 1 (50.0; 1.26–98.74) | 0 (0.0) | 1 (50.0) | 0 (0.0) | 1 (50.0) | 0 (0.0) | 0 (0.0) | 0 (0.0) |
|  | 40 mg QD | 45 | 18 (40.0; 25.70–55.67) | 1 (2.2) | 17 (37.8) | 21 (46.7) | 4 (8.9) | 1 (2.2) | 0 (0.0) | 1 (2.2) |
|  | 20 mg QD | 15 | 7 (46.7; 21.27–73.41) | 1 (6.7) | 6 (40.0) | 6 (40.0) | 2 (13.3) | 0 (0.0) | 0 (0.0) | 0 (0.0) |

CI, confidence interval; CR, complete response; EOD, every other day; IMDC, International Metastatic Renal Cell Carcinoma Database Consortium; NE, not evaluable; ORR, objective response rate; PD, progressive disease; PR, partial response; QD, once daily; RECIST, Response Evaluation Criteria in Solid Tumors; SD, stable disease.
